# A Survey on Workplace Violence Experienced by Critical Care Physicians

**DOI:** 10.5005/jp-journals-10071-23202

**Published:** 2019-07

**Authors:** Nadikuda Sunil Kumar, Kartik Munta, J Raj Kumar, S Manimala Rao, M Dnyaneshwar, Yogesh Harde

**Affiliations:** 1-6 Department of Critical Care Medicine, Yashoda Multi-Speciality Hospital, Somajiguda, Hyderabad, India

**Keywords:** Critical care physicians, Communication, Verbal violence, Workplace violence

## Abstract

**Introduction:**

Workplace violence (WPV) has been defined as, “violent acts including physical assault and threats of assault directed toward personnel at work or on duty”. Healthcare staff are at highest risk of WPV among the professionals and it is more common among the critical care services. Prevalence of WPV among doctors all over the world is around 56–80% and in Indian scenario, it is around 40.8–75%. There is scarcity of studies on WPV among doctors from India. To our knowledge, this is the first of its kind survey conducted to know about the incidence of WPV amongst critical care physicians in India.

**Materials and Methods:**

This survey was conducted after taking due ethical committee clearance amongst critical care physicians attending a critical care conference. The purpose of the study was informed to the participants and a pretested, self-administered, semi-structured questionnaire was distributed among them for their voluntary and anonymous response.

**Results:**

Out of 160 delegates who were given the questionnaire, 118 responses were collected and their forms were analyzed. Maximum responses (84%) received were of age group 20–40 years. Seventy-two percent respondents experienced WPV during their work hours. Most common type of violence reported was verbal violence (67%). Sixty-five percent respondents reported that poor communication was the leading cause of WPV. Due to WPV, most of the respondents (60%) had to change their place and pattern of work. Proper communication (76%) was the most common measure among multiple measures suggested by respondents for avoiding WPV. Eighty-three (98%) respondents opined that conflict management should be part of regular curriculum in medical education.

**Conclusion:**

Improving the communication skills amongst critical care physicians, teaching doctors about conflict management in their regular curriculum of medical education, spreading awareness in public about patient rights and taking initiatives in propagating an idea to “Fight against the diseases and not against the doctors” are the key measures to combat WPV.

**How to cite this article:**

Kumar NS, Munta K, Kumar JR, Rao SM, Dnyaneshwar M, Harde Y. A Survey on Workplace Violence Experienced by Critical Care Physicians. Indian J Crit Care Med 2019;23(7):295–301.

## INTRODUCTION

Violence is a style of communication and conflict resolution, physicians are treated no different from anybody else.^1^ Workplace violence (WPV) has been defined as “violent acts including physical assault and threats of assault directed towards personnel at work or on duty”.^2^ As per OSHA (Occupational safety and health administrative), healthcare staff are at highest risk of WPV among the professionals, with a four-fold high likely chance to get injured and require break from work due to WPV.^3–5^

WPV in hospitals is growing as a global pandemic.^6,7^ Approximate incidence of WPV among doctors all over the world is around 56–80%.^4,8^ Actual extent of the problem is estimated to be substantially higher as the problem remains grossly neglected and under reported.

In developing countries like India WPV has major impact on health sector as this is one of the growing fields, where health expenses are paid by individuals and in recent past, it has been increasing exponentially. Prevalence of WPV amongst doctors in Indian scenario is around 40.8–75%.^9,10^ Violence is more common among the critical care services after psychiatry as they deal with people in highly stressful, emotional and anxious states.^4,5,11,12^ Verbal abuse is the most common type and patient visitors are the most common cause of WPV in hospitals.^13^

WPV is a new upcoming occupational hazard and has significant long lasting effects on healthcare providers.^14^ There exists a dearth of studies on this problem regarding perception of the healthcare providers particularly in young doctors. It remains grossly under researched in India.^4,11^

The purpose of the study is to draw attention toward the issue of violence against critical care physicians, reveal the dimensions of such violence and highlight ill effects of WPV on personal life of doctors. The study also contributes to the measures to be undertaken in addressing this issue and potential recommendations for its prevention in field of “critical care medicine” in India. To our knowledge, this is the first of its kind study conducted on critical care physicians on WPV in India.

## MATERIALS AND METHODS

After taking due ethical committee clearance, the survey was conducted among the critical care physicians attending a critical care conference. Purpose of the study has been informed to the participants and a pretested, self-administered, semi-structured questionnaire was framed under broad sections (mentioned below). The questionnaire was distributed at the registration desk for their voluntary and anonymous response, assurance was given to them concerning confidentiality and responses were collected into a drop box.

**Fig. 1:**
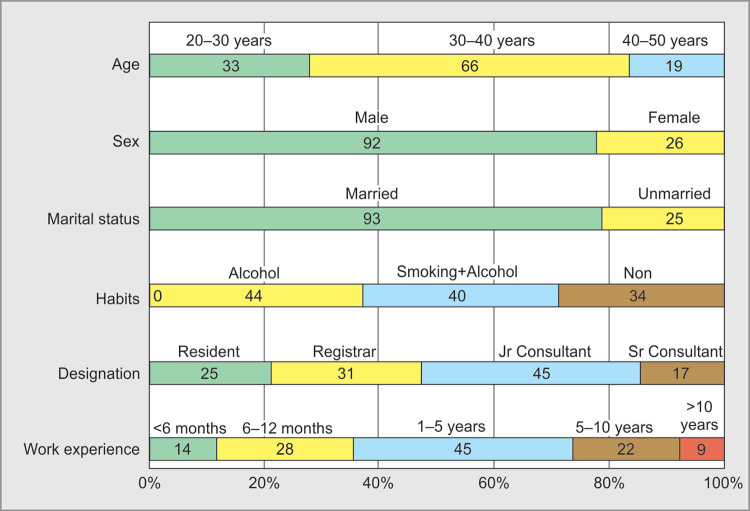
Demographic data

**Questionnaire details:** A total of 30 questions were framed and divided into five main domains.

1. Demographic data – Demographic details with ICU working experience.
2. ICU infrastructure – Type of ICU, facilities available and policies implemented.
3. Violence episode details – Number and type of violence episodes.
4. Violence sequelae – Response of the victim and authorities toward the violence episode.
5. Retrospective analysis – Impact and measures to overcome the violence episode.

Standard definitions adopted from WHO were used to define the types of violence, according to which **verbal violence** was defined as a negative defining statement told to the victim or about the victim, or by withholding any response, thereby defining the target as nonexistent. Similarly **physical violence** was described as the use of physical force against another person or group that results in physical, sexual or psychological harm. This includes beating, kicking, slapping, stabbing, shooting, pushing, biting and pinching.^4^ After collecting the responses, data were entered into Microsoft Excel sheet and analyzed in percentage responses.

### RESULTS

Out of 160 delegates who were given the questionnaire, 118 responses were collected and analyzed.

### Demographic Data

Maximum respondents were in the age group of 20–40 years (99; 84%), with a working experience of less than five years in the field of critical care medicine 87 **(74%)**. Of all the respondents, (92; 78%) were males and 26 **(22%)** were females. Ninety-three **(79%)** respondents were married (Fig. 1).

### ICU Infrastructure

**Fig. 2:**
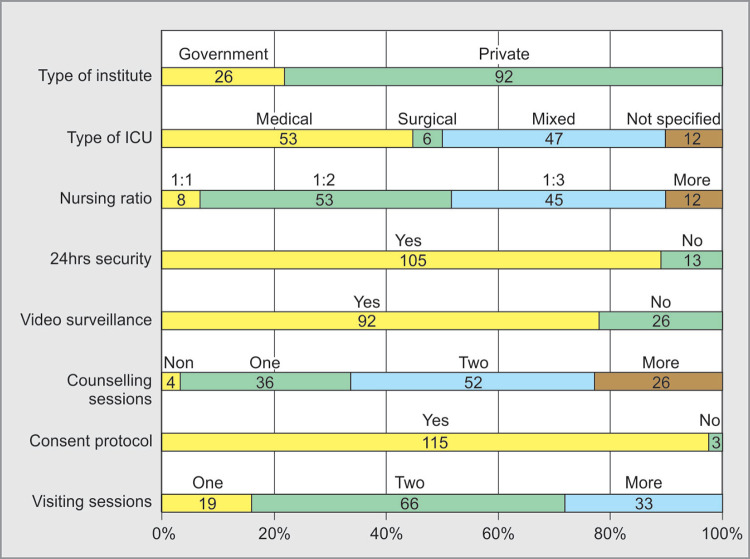
ICU Infrastructure

Maximum respondents were from private hospitals i.e. 92 **(78%)**, mostly from medical ICU's i.e. 53 **(45%)** and 47 **(40%)** respondents were from mixed ICU's. Median number of beds were 15, with a nursing ratio around 1:2 (53; 45%) to 1:3 (45; 38%). Most of the ICU's (105; 89%) had 24hours security cover, performed counseling (114; 97%) and practice consent protocols (115; 97%) (Fig. 2).

### Violence Episode Details

**Fig. 3:**
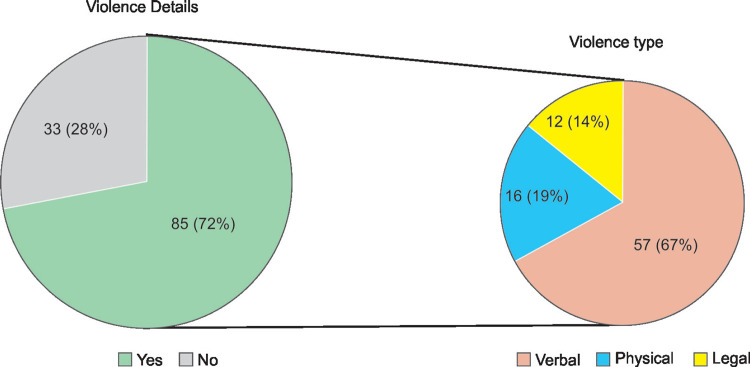
Violence details

**Fig. 4:**
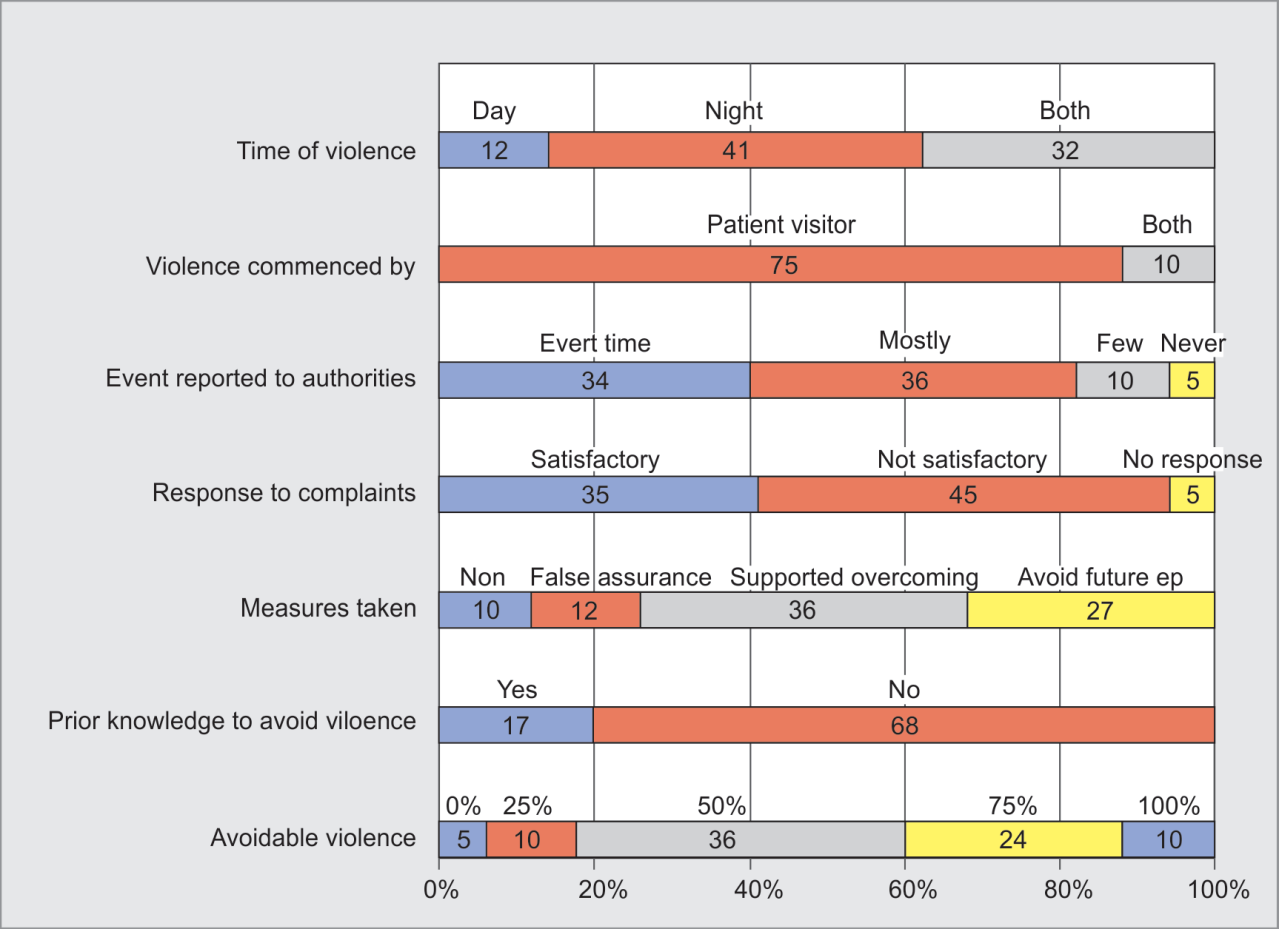
Violence details and sequela

Among 118 respondents, 85 **(72%)** experienced WPV during their ICU work hours. Maximum episodes were experienced during night times when working staff was less. Most common type of violence was verbal 57 **(67%)** and in maximum episodes patient visitors were the cause of violence 75 **(88%)**. Most of the events i.e. 70 **(85%)** were reported to concerned authorities and the response was non-satisfactory according to 45 **(53%)** respondents (Figs 3 and 4).

### Causes of Violence

**Fig. 5:**
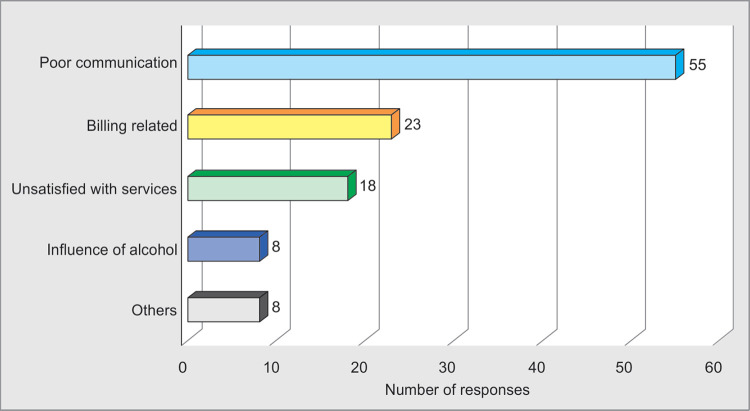
Causes of violence

Poor communication (**65%;** 55 out of 85) was the leading cause of WPV. Billing related disputes were **27%** (23/85), dissatisfaction regarding medical services were **21%** (18/85) among other major causes. Among respondents, only 17 **(20%)** had prior knowledge and experience of managing such WPV episodes (Figs 4 and 5).

### Effects of Violence

**Fig. 6:**
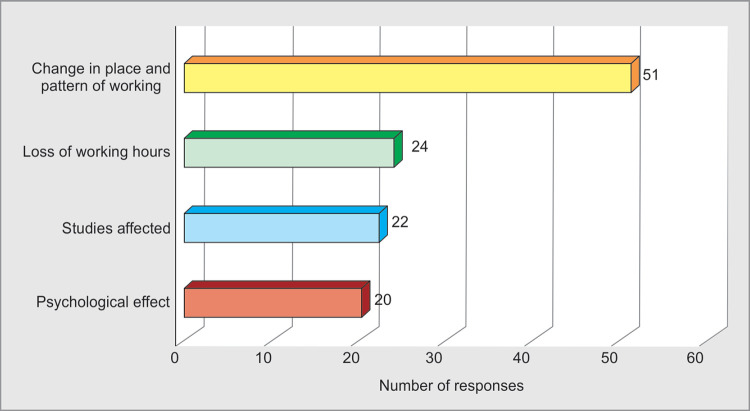
Impact of violence

Due to WPV most of the respondents had to change their place and pattern of work **60%** (51/85), and there were loss of working hours **28%** (24/85). The respondents opined that it had affected their education **26%**(22/85) and had profound psychological impact **23%**(20/85) on them (Fig. 6).

### Remedial Measures Suggested

Most of the respondents **70%** (60/85) felt that maximum number of episodes **(50–70%)** which happened were avoidable. Remedial measures like proper communication **76%** (65/85), by improving security **49%** (42/85), infrastructure **47%** (40/85), ensuring vigilant monitoring **29%** (25/85), increasing responsibilities of the hospital authorities **29%** (25/85) were suggested by the respondents for avoiding WPV.

**Fig. 7:**
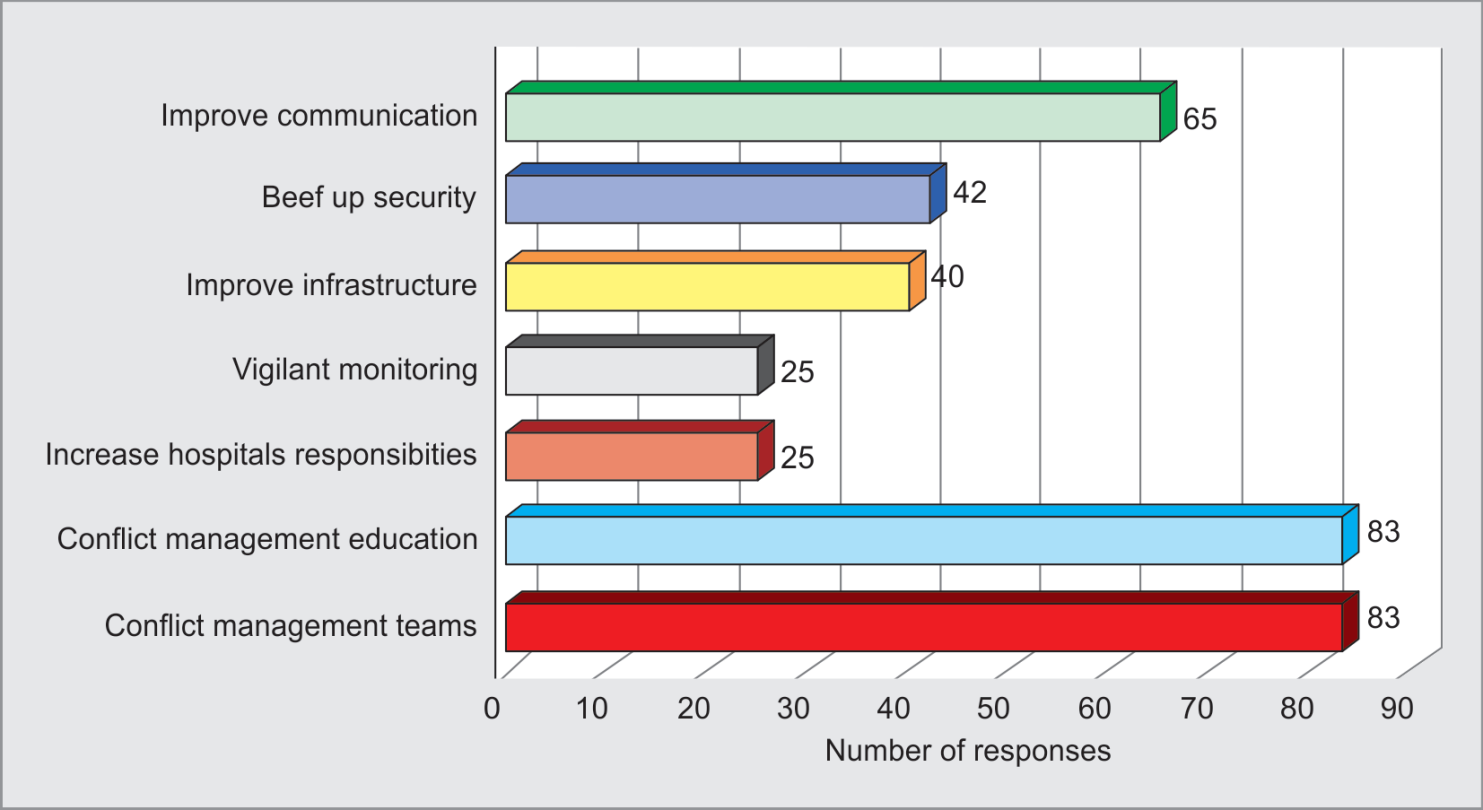
Remedial measures

**Ninety-eight percent** (83/85) respondents felt that conflict management should be a part of their regular curriculum of medical education and conflict management teams should be formed in hospitals to avoid, advise, support and overcome episodes of WPV (Figs 4 and 7).

### Limitations of the Study

Survey was conducted amongst critical care physicians attending a critical care conference hence generalization of the findings cannot be done. Participant's reported violence, relevant exposure which had no time frame. Hence there is a potential for selection and recall bias. Maximum respondents were young and inexperienced, which may lead to biased results. Despite these limitations our study has provided an insight into the incidence of WPV among critical care physicians, highlighting the potential risk factors, impact on their personal life and the preventive measures to be taken to avoid such episodes.

## DISCUSSION

Annual incidence of WPV is four times more in healthcare (8 serious cases per 10,000 full time employees) as compared to all other professions (2 per 10,000)^2,8^ and working in healthcare institution is 16 times more risky than working in another business. Nurses working in psychiatric department are third group of professionals most exposed to violence after prison guards and police officers.^12^

During 1980–1990, hundred healthcare workers died as a result of violence in USA; a survey in 170 university hospitals over 5 years revealed 57% of all employees in emergency department had been threatened with weapon.^15,16^ In 2008, a survey conducted on 600 doctors of Britain showed 1/3rd to be victims of violence and half among them did not report the incident.^7^ In Israel, 70% of emergency department physicians and 90% of supporting staff working had experienced violence, amongst them verbal abuse was most common.^7^ In China, incidence of non-physical violence is 68–76% and physical is around 8–35%.^6^ In Pakistan incidence of WPV in doctors is around 74%.^17^ Factors differ from developed to developing countries where education and low socioeconomic status play a major role along with poor infrastructure.^6^

Indian Medical Association has reported that 75% doctors had experienced violence and 82.7% had profound work stress.^7,14,18^ Almost half of the violent incidents occur in critical care units, wherein the intensivists working here face such episodes almost daily. Such incidents are more common in government hospitals where workforce is less and infrastructure is poor.^19^ Our study has shown an incidence of WPV to be 72% amongst which 22% respondents were from government sector of whom maximum experienced WPV.

In the Anglo and anglicized regions of the world .i.e. United Kingdom, Ireland, New Zeland, Australia, United States and Canada, physical violence is reported more commonly. Bullying is common in the Middle East.^8^ Very few studies have been undertaken in Asia, Indian subcontinent, out of which recent studies are from China.^18–20^ Most common type of violence is verbal abuse and least common is physical violence among all departments in Indian context.^4,5,21^ Most incidents occurred during morning or night hours and took place in hospital premises.^4^ Highest rate of incidents were reported in the evening between 4–8 pm.^12^ In our study, verbal abuse (67%) was most common type of violence. WPV was most commonly initiated by patient visitors (88%) during night times (48%). Whereas 38% experienced it both day and night.

Healthcare WPV is a serious emerging threat to the patient-doctor mutual relationship all over the world. It has evolved in to a prominent challenge for medical profession.^4,14^ Most of the studies done worldwide on WPV proved, poor communication to be the most common cause of violence (Table 1). A study conducted in public hospital in Mumbai, India, showed that most doctors and almost all patients felt that many problems would be resolved if patients were treated with more respect. Residents are usually entrusted with delicate tasks as breaking the bad news or events happening in operating or emergency room. Seniors should deal with serious issues like breaking the bad news and explaining the untoward events happening in hospitals.^23,24^ In our study, poor communication (65%), billing related issues (27%) and dissatisfaction with services (21%) were the common causes of WPV among respondents. Amongst the respondents only 20% had prior knowledge and experience of handling WPV episodes.

**Table 1:** Causes, effects and remedial measures of workplace violence

Causes^4,5,7,12^ Poor communication Long waiting periods, delays in medical and nursing care provision Violation of visiting hours Denial of patient's admission in the hospital, financial factors Patients dissatisfaction with nursing/medical care, rude behaviour of staff Sudden death of patient Poor conflict resolution skills among physicians Drug addiction and Psychological problems among patient visitors Negative role of media, politicians, public and mob psychology Insufficient health budget and gaps in judicial system
Effects^4,5,12,14,22^ Increased dissatisfaction toward profession Demoralization, loss of confidence Anger, frustration, irritability Fear, anxiety, sleep disturbances, headache Short-term and long-term effects on physical performance (refrain from working alone, abstinence from work) Short-term and long-term psychological effects (depression) Short-term and long-term effects on professional performance (change in attitude of working) Physical disabilities Stress disorders (PTSD) Discouraging their progeny from taking the profession
Measures^4,5,7,11,14^ Good communication skills with due respect System to give regular information to patient visitors to relieve their anxiety Improved security and infrastructure Full time video surveillance and panic alarms Educating conflict management skills Encourage reporting of the events to the concerned authorities Violence prevention and control committees in hospitals, insure against mob violence and damage of property Public must be taught about public rights through display boards Health insurances and health budget strengthening Strong judiciary support

WPV has a negative impact on the physical and psychological well being.^5^ WPV affects the health of the doctor in long run via psychological stress, which leads to sleep disturbances and quality of care.^4,14^ A study conducted in Canada showed 73% respondents had fear of treating patients due to WPV. Seventy-four percent reported reduced job satisfaction and 67% had a job change^22^ (Table 1). In our study, 60% respondents reported change in their place and pattern of working, 28% lost working hours, 26% had effect on their studies and 23% had psychological impact (Fig. 6).

Though doctors are the victims of WPV, society as a whole has to pay the cost due to change in the attitude of doctors toward medical profession and patients, which gets transmitted horizontally and vertically.^14^ A study conducted in 2002 by Jiao et al. revealed only 11% Chinese doctors wanted their children to join medical profession, but in 2011 this further reduced to 7%, as a consequence recruitment and retention of doctors have become major challenge for the Chinese healthcare system.^25^ Similar sentiments are being echoed in Indian subcontinent also.^19,26^

Violence is routinely under reported in health sector because the perception among the healthcare workers is that, violence is an expected part of their job and fear the response they may receive when such events are reported. In the present study a meager 40% reported WPV events to the concerned authorities every time.

A cross sectional study in China concluded three types of remedial measures for WPV- individual, organisational and social training combined with legal and security measures.^27^ It has been emphasized that lack of professional training for handling violent incidents may be the reason for less experienced professionals to become victims. Looking for indicators of violent behavior such as Staring looks, Tone and violence in voice, Anxiety, Mumbling, and Pacing (STAMP behavior) are few predictors^7^ (Table 1). In our study, respondents felt communication (76%) plays an important role, and improving security (49%) and infrastructure devolopment (47%) would help. Other measures advised were vigilant management and increasing hospital administration responsibility in avoiding such acts (Fig. 7).

Physical exercise, sleep, company of family, friends and colleagues were the most useful coping strategies.^22^ Doctors who were given lessons in communication could manage most potentially violent situations.^23^ Medical education and CME's should include this topic giving appropriate emphasis.^28^ Indian studies in the past emphasized on legal measures, security beef up and increasing number of doctors and other staff have been recommended.^4,5^ Only installing security personnel may not yield long lasting results, in fact it may worsen some situations.^6^ In our study almost 98% advised that conflict management education should be the part of medical education curriculum and conflict management teams to be formed in hospitals (Table 1 and Fig. 7).

According to the OSHA WPV can be prevented or the risk at least minimized, when employers take the necessary precautions. OSHA advocated an interdisciplinary approach to WPV prevention and implementation of a **“Zero tolerance policy”** to help achieve goal of resolving WPV. Uploading videos for public education, forming and empowering hospital violence prevention and control committee, improving doctor-patient mutual relations, improving security and using hi-tech security measures have been few of their suggestions.^14^

Few hospitals in UK used a **“Zero tolerance policy”** with green, yellow and finally red warning cards according to the type of violence which penalized aggressive behavior by transfer to another hospital.^11,19^ Nineteen states in India have dedicated nodal agencies and institutions in handling incidents of WPV.^7^ States like Odisha, Maharashtra and Kerala have passed laws for punishment of WPV for medical services in recent past.^4,13^ To prevent violence in hospitals in India, **“Hospital Protection Act”** was passed in 2008 where persons engaged in violence are liable to be imprisoned for 3–10 years under legislation.^12^

## CONCLUSION

Improving the communication skills among doctors is the need of the hour, this can be improved by including conflict management in regular curriculum of medical education and conducting regular CME programs. As WPV is highest in emergency and critical care department, conflict management training should be more stressed while training physicians in these departments. Increasing the responsibilities of hospital administration, enhancement of security in the night times, spreading awareness in public about patient rights, strengthening the medical insurance and judiciary system play an integral role in curtaining this menace in India. Let's all spread the word to “**Fight against the diseases and not against the doctors**”.
